# Association of wrist circumference with metabolic dysfunction associated fatty liver disease in the KADEM cohort

**DOI:** 10.3389/fendo.2025.1580209

**Published:** 2025-08-07

**Authors:** Fahad Al-Ajmi, Mohamed Abu-Farha, Ahmed N. Albatineh, Zahraa Ali, Abdullah AL-Enezi, Mohammed Alterki, Rashed Alhammad, Retaj AlHarbi, Mohamed Shehab, Muhammad Abdul-Ghani, Jehad Abubaker, Fahd Al-Mulla

**Affiliations:** ^1^ Department of Biochemistry and Molecular Biology, Dasman Diabetes Institute, Dasman, Kuwait; ^2^ Department of Translational Research, Dasman Diabetes Institute, Dasman, Kuwait; ^3^ Department of Biostatistics and Health Data Science, College of Health, Lehigh University, Bethlehem, PA, United States; ^4^ Department of Pharmacology, Faculty of Medicine, Kuwait University, Kuwait City, Kuwait; ^5^ Division of Diabetes, University of Texas Health Science Center, San Antonio, TX, United States

**Keywords:** wrist circumference, MAFLD, T2D, obesity, CAP

## Abstract

**Introduction:**

Metabolic dysfunction-associated fatty liver disease (MAFLD) is linked to metabolic dysfunction and to an increased risk of cardiovascular diseases. The early detection of individuals at high risk of MAFLD is essential for timely interventions. This study explores the association between wrist circumference and MAFLD among participants in the Kuwait Adult Diabetes and Epidemiological Multidisciplinary (KADEM) program, aiming to evaluate wrist circumference as a potential noninvasive diagnostic marker.

**Materials and methods:**

This study included 449 participants assessed for MAFLD using FibroScan^®^. The MAFLD stages were classified according to the controlled attenuation parameter (CAP) score into four groups: normal (<238 dB/m), S1 (238–260 dB/m), S2 (261–290 dB/m), and S3 (>290 dB/m). Participants underwent routine clinical blood tests, and measurements of body mass index (BMI) and wrist circumference were recorded. Multiple logistic regression was used to evaluate the discriminative ability of wrist circumference and other covariates in predicting high CAP scores, with the area under the receiver operating characteristic (ROC) curve (AUC), the sensitivity, and the specificity reported.

**Results:**

The cohort included 184 (41.5%) normal, 77 (17.4%) S1, 81 (18.3%) S2, and 101 (22.8%) S3 participants. Wrist circumference was significantly different across groups: 16 cm for normal, 17 cm for S1 and S2, and 17.2 cm for S3 (*p* < 0.001). A strong correlation between wrist circumference and MAFLD was found (*r* = 0.328, *p* < 0.001). Wrist circumference was positively correlated with the triglyceride (TG), alanine transaminase (ALT), and aspartate aminotransferase (AST) levels and negatively correlated with high-density lipoprotein (HDL; *p* < 0.05). Adjusted analysis showed that gender, wrist circumference, ALT, and TG were significantly associated with high CAP scores. A multiple logistic regression model including these variables discriminated 76.3% of the subjects, with 69.4% sensitivity and 73.4% specificity.

**Conclusion:**

Wrist circumference is a potential noninvasive marker for the identification of individuals at high risk of MAFLD, representing a cost-effective screening tool for early detection. Further research is needed to confirm its clinical utility.

## 1 Introduction

Metabolic dysfunction-associated fatty liver disease (MAFLD) is a recently adopted terminology to replace the former term, non-alcoholic fatty liver disease (NAFLD). It provides a more inclusive and accurate perspective on the disease, linking it to the presence of metabolic dysfunction rather than the absence of alcohol use (1). A proposed criterion suggested that a positive diagnosis of MAFLD must be supported by either histological or imaging evidence for liver steatosis and the presence of either body mass index (BMI) >25, type 2 diabetes mellitus (T2DM), or markers of metabolic dysregulation (2). Furthermore, obesity, type 2 diabetes, hypertension, hyperlipidemia, metabolic dysregulation, and genetic influences are metabolic factors that contribute to the accumulation of fat in hepatic cells and, without proper management, can lead to severe liver damage in conditions such as cirrhosis, fibrosis, and hepatocellular carcinoma. Moreover, metabolic factors can increase the risk of developing cardiovascular diseases. Currently, MAFLD affects approximately 39% of the global population, rendering it one of the most prevalent liver disorders in our present day (3–5). Given the widespread prevalence of MAFLD and its typically asymptomatic nature until the advanced stages, there is a critical need for improved diagnostic strategies to enable early detection and interventions (1, 4, 6).

The gold standard for determining fat accumulation, inflammation, and fibrosis, as well as for diagnosing MAFLD, is liver biopsy. Furthermore, imaging methods such as MRI, FibroScan, and ultrasound are authorized for the diagnosis of MAFLD. Numerous studies have shown that the biomarkers for inflammation and fibrosis, including serum albumin (ALB), alkaline phosphatase (ALP), alanine transaminase (ALT), aspartate aminotransferase (AST), resistin, leptin, interleukin 6 (IL-6), and tumor necrosis factor alpha (TNF-α), can help predict inflammation and fibrosis and could be useful in risk classification and MAFLD diagnosis (7–9).

Wrist circumference has emerged as a promising anthropometric marker due to its strong association with metabolic traits, including body fat accumulation, insulin resistance, and obesity. Unlike BMI or waist circumference, wrist circumference may offer a practical advantage by reflecting the central and peripheral fat distribution while being easy to measure in both the clinical and field settings. Previous studies have explored the correlation between arm circumference (AC) and metabolic syndrome, insulin resistance, and NAFLD (10–12). For instance, Zou et al. (12) reported a positive association between AC and the risk of MAFLD, while Wang et al. (13) demonstrated a significant relationship between AC and liver steatosis/fibrosis in patients with MAFLD. In addition, wrist circumference has been linked to the progression from metabolically healthy to unhealthy obesity, particularly in women (11). However, our study makes a unique contribution by evaluating wrist circumference as a predictor of controlled attenuation parameter (CAP)-quantified MAFLD in a Kuwaiti cohort, a population that has been underrepresented in prior research. Furthermore, our work aimed to introduce a safe, simple, and noninvasive screening tool for the identification of individuals at high risk of MAFLD.

Given its potential to serve as a surrogate marker for metabolic dysfunction, we propose wrist circumference as a noninvasive and cost-effective tool for predicting the risk of MAFLD. This approach may facilitate the early identification of individuals with or who are at risk of hepatic steatosis, thereby supporting timely interventions and reducing the burden of MAFLD and its cardiovascular complications (7, 11). Based on this rationale, we hypothesized that individuals with larger wrist circumference values would exhibit higher levels of hepatic steatosis and an increased risk of metabolic comorbidities. To test this hypothesis, we measured the wrist circumference in a cohort of 449 participants from the Kuwait Adult Diabetes and Epidemiological Multidisciplinary (KADEM) study and examined its association with fatty liver as assessed using FibroScan^®^.

## 2 Materials and methods

### 2.1 Study population

KADEM is a study conducted at the Dasman Diabetes Institute (DDI). The study was approved by the DDI Ethical Committee (study no. RA2019–030 and registered at clinicaltrials.gov, NCT06115876) and was conducted in accordance with the ethical framework of the Helsinki Declaration. Random sampling of the Kuwaiti population with proportional representation from each of the seven governorates was conducted for participant recruitment. A list of Kuwaiti residents, complete with their unique identification codes, was provided by the National Public Authority of Civil Information. A stratified random sampling technique was employed for the selection of survey participants from this resident list. The survey design was adapted from the WHO STEPwise approach to surveillance (STEPS) methodology. All participants signed a consent form before participation in the study. A total of 449 participants were enrolled in KADEM. Individuals suffering from any infection and those aged younger than 18 or older than 65 years were excluded from the study. In addition, data on the participants’ hepatitis status and medication use were not collected, which may have introduced residual confounding and should be addressed in future studies. MAFLD was assessed by a trained nurse using vibration-controlled transient elastography (VCTE) with a FibroScan^®^ device. Liver stiffness was assessed with the liver stiffness measurement (LSM) values in kilopascals, with the severity of fibrosis categorized as follows: <6 (F0, normal), 6–8 (F1/2), and 8–12 (F3). The severity of hepatic steatosis was assessed based on the CAP values (in decibels per meter), which was graded as <238 (normal), 238≤ (S1) ≤259, 260≤ (S2) ≤290, and over 290 (S3). To assess the clinical variables, the participants were asked to fast for at least 10 h before collection of blood samples to measure the lipid and glycemic profiles, including triglycerides (TG) hemoglobin A1c (HbA1c), fasting plasma glucose (FPG), total cholesterol (TC), high-density lipoprotein (HDL), and low-density lipoprotein (LDL). Siemens Dimension RXL chemistry analyzer was used for the glucose and lipid profiles and the Variant^TM^ device used for the HbA1c levels.

Anthropometric measures were also recorded, including the age, gender, and BMI, as well as clinical laboratory tests evaluating the lipid profile, the liver function test, and glucose. Wrist circumference was measured with the participants standing in a relaxed, upright position, with their arms resting naturally at their sides and the palms facing inward. Trained nurses conducted the measurements using a constant-tension (non-stretchable) tape measure to ensure consistency and accuracy across participants. The measurement site was defined as the midpoint between the prominent ulnar styloid process and the radial head on the dorsal aspect of the wrist. The tape was wrapped horizontally around the wrist at this midpoint, ensuring that it was snug against the skin without compressing soft tissue or causing indentation, which was assessed to evaluate its correlation with MAFLD. BMI was calculated by dividing the study subjects’ weight in kilograms, measured using an electronic weighing scale, divided by the square of their height in meters, measured using portable inflexible height measuring bars (BMI = kg/m^2^). Individuals were classified as being underweight (<18.5 kg/m^2^), normal weight (18.5–24.9 kg/m^2^), overweight (25–29.9 kg/m^2^), or obese (>30 kg/m^2^).

### 2.2 Blood processing

Blood samples were collected in EDTA tubes and centrifuged at 400 × *g* for 10 min at room temperature to separate the plasma. The plasma was further centrifuged at 800 × *g* for 10 min to obtain a clear supernatant, which was aliquoted into fresh tubes. Plasma samples were stored at −80°C for subsequent tests.

### 2.3 Statistical analysis

The statistical software STATA version 14 (STATA Corp, 2023), the Statistical Software for Social Sciences (SPSS) (version 29; IBM Corp., Armonk, NY, USA), and R statistical software (R Core Team) were used for data analysis. Data were imported and assessed for abnormalities and then coded for descriptive purposes. Continuous variables were reported as the mean and standard deviation (SD) if normality was satisfied; otherwise, the median and the interquartile range (IQR) were reported. Two-sample *t*-tests were used to examine the equality of the means if the normality of two groups was satisfied; otherwise, the Mann–Whitney *U* test was implemented. To test for the association between two categorical variables, Pearson’s chi-square test of independence was utilized if the expected cell counts for ≥80% of the cells were more than 5; otherwise, Fisher’s exact test was applied. Spearman’s correlation coefficient was implemented due to the presence of outliers to measure the strength of the linear relationship between two continuous variables.

The main outcome was the CAP score, which, according to the steatosis grade, has the following categorization: normal (<238 dB/m), S1 (238–259 dB/m), S2 (260–290 dB/m), and S3 (>290 dB/m). As our goal was to estimate the odds of patients with higher CAP scores, a two-level outcome (normal ≤ 290 dB/m and high > 290 dB/m) was derived and implemented in the analysis.

A multiple logistic regression model was utilized to model the association between the CAP score (binary outcome: 0 = normal, 1 = high) and several covariates. The odds ratios (ORs) with their corresponding 95% confidence intervals (95%CIs) were reported. The Hosmer–Lemeshow test was used to assess the goodness of fit of the model.

To assess the predictive ability of the multiple logistic regression model as a discriminating tool, the area under the receiver operating characteristic (ROC) curve (AUC) was reported. Furthermore, the sensitivity, the specificity, and the cutoff value for the main exposure of the logistic regression model were reported based on the optimal cutoff value produced by the Youden index. All tests were two-tailed, and the level of significance was set at 5%.

## 3 Results

### 3.1 Demographic and clinical characteristics of participants by CAP scores

**Figure 1:**
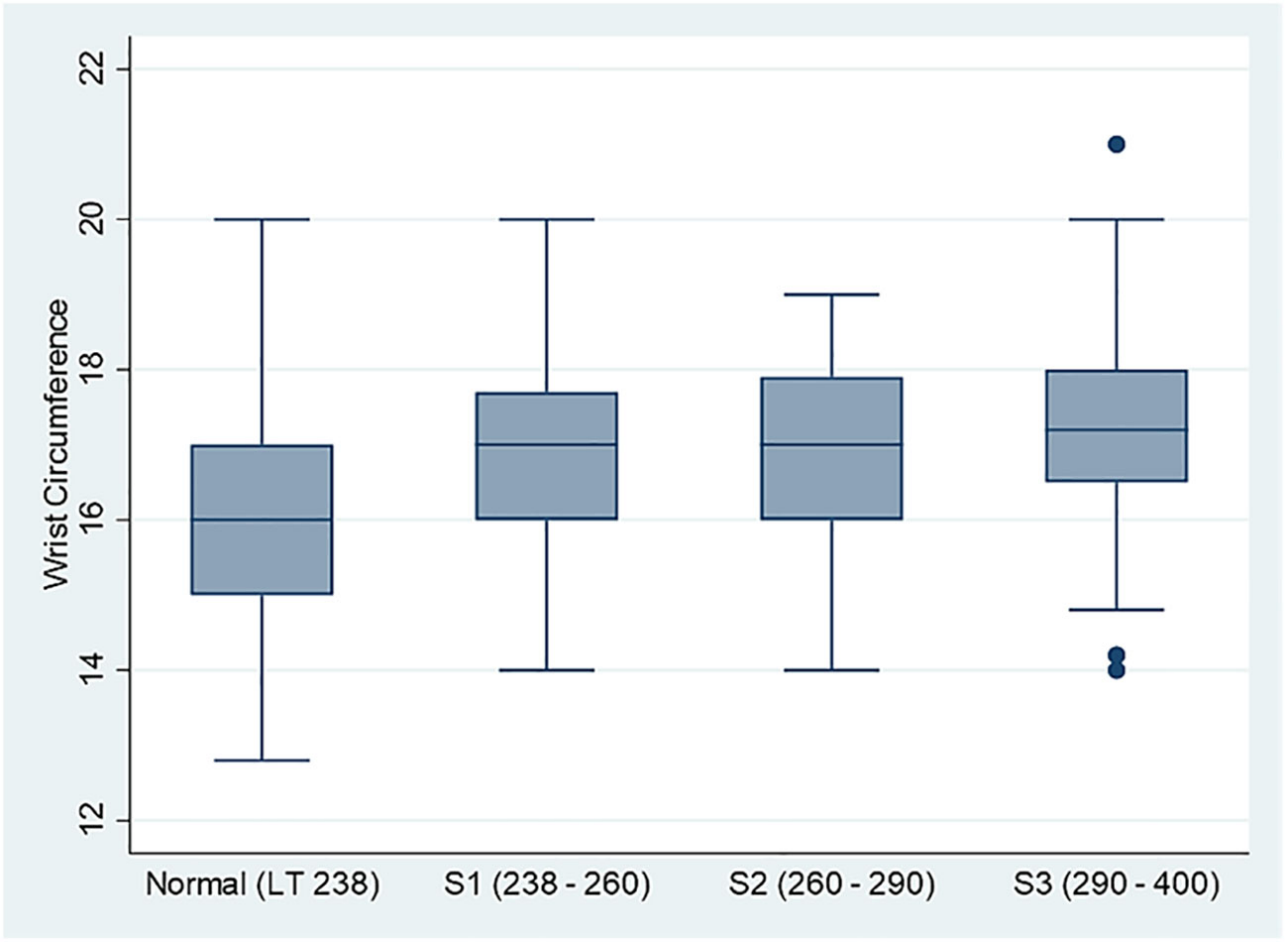
Comparison box plot for the distribution of the wrist circumference measurements across four controlled attenuation parameter (CAP) score categories (*N* = 449).

Our cohort comprised 449 subjects: 184 (41.5%) were classified as normal based on the CAP score, the S1 level had 77 (17.4%) participants, the S2 level had 81 (18.3%), and the S3 level had 101 (22.8%) participants. **Figure 1** illustrates the distribution of the wrist circumference values, where individuals with normal values had an average circumference of 16 cm (IQR = 2.0), while those classified as S1 and S2 averaged 17 cm (IQR = 1.8). Participants at the S3 level showed a slightly higher average of 17.2 cm (IQR = 1.5). With a *p* < 0.001, using a hypothesis test summary, the data indicated significant variations in the wrist circumference values across the different CAP score cohorts. Subjects classified as normal had an average age of 44 years. In contrast, the average age for participants in the S1 level was 51 years, those in the S2 level was 53 years, and those in the S3 level was 50 years. In terms of BMI, subjects with normal values recorded a median BMI of 26.1 kg/m^2^ compared with 28.4 kg/m^2^ for the S1 group, 28.9 kg/m^2^ for the S2 group, and 32.6 kg/m^2^ for those in the S3 category.

The data in **Table 1** present the demographic and clinical characteristics of the participants by CAP scores, where a score ≤290 is considered a normal score and a score <290 is regarded as high. The male percentage (26.1%) was higher than the women (18.3%) in the high CAP score group, while in the normal group, there was a higher female percentage (81.7%) than men (73.9%), with a marginal significant association between gender and the CAP score (*p* = 0.053). Significant differences in the median values for wrist circumference, BMI, waist circumference, waist-to-hip ratio, and the ALT, AST, and HDL levels indicate that higher CAP scores are associated with poor metabolic profiles. For instance, the median BMI in the high CAP score group was notably higher at 32.6 compared to the 27.6 in the normal group (*p* < 0.001). The same holds for waist circumference and the waist-to-hip ratio.

**Table 1:** Descriptive analysis of the controlled attenuation parameter (CAP) score categories normal (≤290) and high (>290) with certain biomarkers (*N* ^a^ = 443).

Covariate	CAP score category		*p*-value^b^
	Normal (<290)	High (≥290)	
Gender, *n* (%)			0.053
Men	170 (73.9)	60 (26.1)	
Women	174 (81.7)	39 (18.3)	
Age (years)	48.0 (16)	50 (10)	0.194
Wrist circumference (cm)	16.8 (1.8)	17.2 (1.5)	**<0.001**
BMI (kg/m^2^)	27.6 (6.7)	32.6 (7.8)	**<0.001**
Waist circumference (cm)	97 (19)	111 (13.8)	**<0.001**
Waist-to-hip ratio	0.912 (0.11)	0.965 (0.08)	**<0.001**
TC (mmol/L)	5.1 (1.51)	5.3 (1.15)	0.181
LDL (mmol/L)	3.2 (1.3)	3.3 (1.15)	0.113
HDL (mmol/L)	1.49 (0.45)	1.32 (0.33)	**<0.001**
TG (mmol/L)	0.90 (0.62)	1.31 (0.95)	**<0.001**
ALT (U/L)	27 (14)	37 (27)	**<0.001**
AST (U/L)	20 (7)	21 (14)	**0.007**

Values reported are the counts (percentage) for gender and the median (interquartile range) for all continuous variables. *P*-values are included to demonstrate significant differences.

*TC*, total cholesterol; *LDL*, low-density lipoprotein; *HDL*, high-density lipoprotein; *TG*, triglycerides; *ALT*, alanine transaminase; *AST*, aspartate aminotransferase.

a Counts may not add up to *N* due to a few missing values.

b The *p*-values for the continuous variables were calculated using the Mann–Whitney *U* test due to the non-normality of at least one group, except for gender, where Pearson’s chi-square test of independence was used.

Values in bold indicate statistically significant differences (p < 0.05).

### 3.2 Correlation analyses of various markers and the CAP score

**Figure 2:**
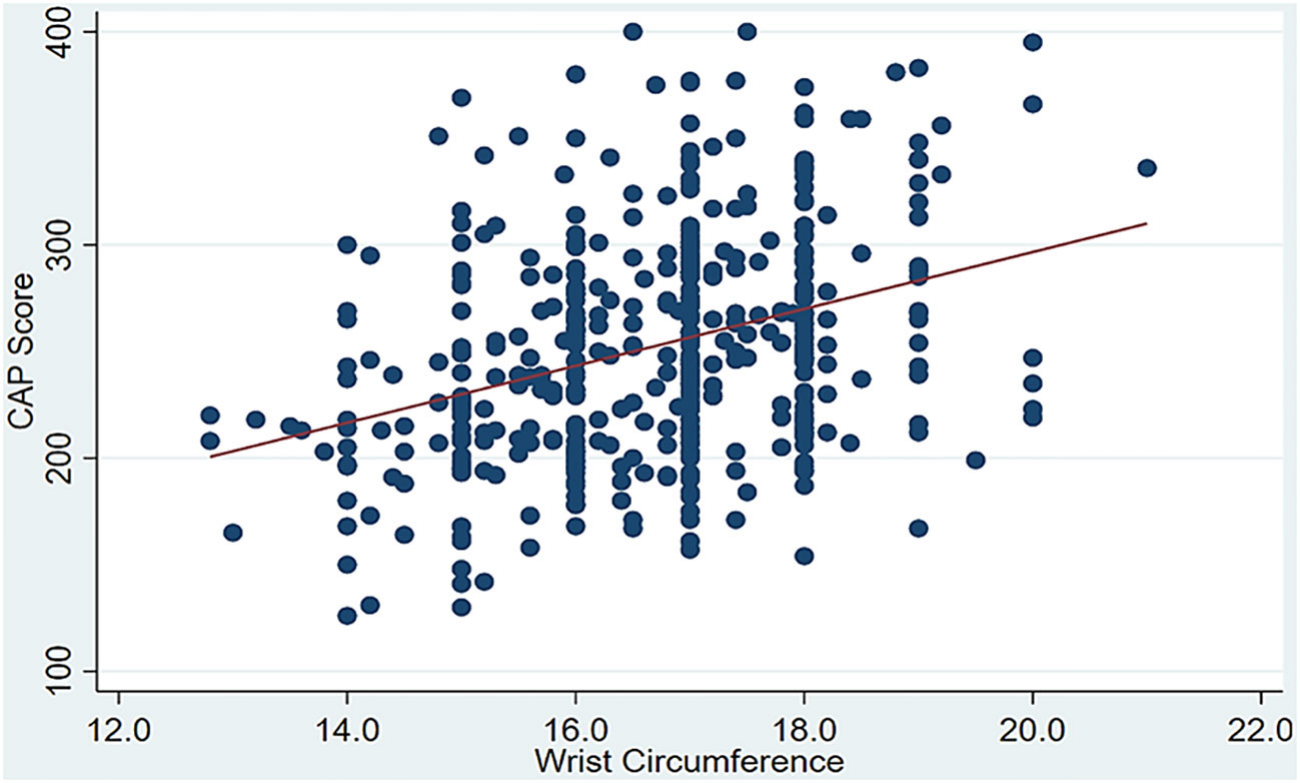
Significant positive correlation between the controlled attenuation parameter (CAP) score and wrist circumference.

To shed more light on the strength of the linear relationship between the CAP score as a continuous covariate and several anthropometric and lipid measurements, the results using Spearman’s rank correlation indicated that the CAP score was directly and significantly associated with age (*ρ* = 0.249, *p* < 0.001), wrist circumference (*ρ* = 0.328, *p* < 0.001) (**Figure 2**), BMI (*ρ* = 0.458, *p* < 0.001), TC (*ρ* = 0.120, *p* < 0.012), LDL (*ρ* = 0.132, *p* < 0.006), TG (*ρ* = 0.455, *p* < 0.001), ALT (*ρ* = 0.352, *p* < 0.001), and AST (*ρ* = 0.157, *p* < 0.001). Only HDL (*ρ* = −0.308, *p* < 0.001) was inversely and significantly associated with the CAP score. Furthermore, the point-biserial correlation indicated that men tended to have lower CAP scores than women (*ρ* = −0.149, *p* = 0.002), as detailed in **Table 2**.

**Table 2:** Spearman’s correlations between gender, age, wrist circumference, BMI, waist circumference, waist-to-hip ratio, and biomarkers with the controlled attenuation parameter (CAP) score.

Covariate	Correlation (*p*-value)	Covariate	Correlation (*p*-value)
Gender	−0.149 (**0.002**)	LDL (mmol/L)	0.132 (**0.006**)
Age (years)	0.249 (**<0.001**)	TG (mmol/L)	0.455 (**<0.001**)
Wrist circumference (cm)	0.328 (**<0.001**)	HDL (mmol/L)	-0.308 (**<0.001**)
BMI (kg/m^2^)	0.458 (**<0.001**)	ALT (U/L)	0.352 (**<0.001**)
TC (mmol/L)	0.120 (**0.012**)	AST (U/L)	0.157 (**0.001**)
Waist circumference (cm)	0.507 (**<0.001**)	Waist-to-hip ratio	0.391 (**<0.001**)

TC, total cholesterol; LDL, low-density lipoprotein; HDL, high-density lipoprotein; TG, triglycerides; ALT, alanine transaminase; AST, aspartate aminotransferase.

Values in bold indicate statistically significant differences (*p* < 0.05).

For the investigation of the association between the CAP score and other covariates, the multivariable logistic regression model indicated that gender, wrist circumference, ALT, and TG were independently and significantly associated with high CAP scores. In the adjusted model, the results indicated that women have more than twice the odds [adjusted odds ratio (AOR) = 2.211, 95%CI = 1.190–4.107] of having a high CAP score compared with men after adjusting for other covariates. Furthermore, a 1-cm increase in wrist circumference would incur a 57.1% increase in the odds of having a high CAP score (AOR = 1.573, 95%CI = 1.243–1.991). This simply means that, for every additional centimeter in wrist circumference, the likelihood of having a high CAP score rises by more than half compared with an individual with a smaller wrist circumference. In addition, a 1-U increase in ALT would incur a 3.6% increase in the odds of having a high CAP score (AOR = 1.036, 95%CI = 1.021–1.051). Finally, a 1-U increase in TG would incur an 85.6% increase in the odds of having a high CAP score (AOR = 1.856, 95%CI = 1.351–2.550). The details are presented in **Table 3**. Finally, according to the results of the Hosmer–Lemeshow test, the chi-square value was 4.499, with 8 degrees of freedom, leading to a *p*-value of 0.81, which indicates that the model fitted the data well.

**Table 3:** Association between the controlled attenuation parameter (CAP) score and the anthropometric covariates using multiple logistic regression analysis (*N* = 449).

Covariate	Univariable analysis		Adjusted analysis	
	OR (95%CI)	*p*-value	AOR (95%CI)	*p*-value
Gender				
Men	Reference	0.051	Reference	**0.012**
Women	0.635 (0.403–1.001)		2.211 (1.190–4.107)	
Wrist circumference (cm)	1.527 (1.274–1.831)	**<0.001**	1.573 (1.243–1.991)	**<0.001**
ALT (U/L)	1.040 (1.026–1.054)	**<0.001**	1.036 (1.021–1.051)	**<0.001**
TG (mmol/L)	1.744 (1.311–2.320)	**<0.001**	1.856 (1.351–2.550)	**<0.001**

*OR*, odds ratio; *AOR*, adjusted odds ratio; *PV*, probability value; *TG*, triglycerides; *ALT*, alanine transaminase.

Values in bold indicate statistically significant differences (*p* < 0.05).

### 3.3 Multiple logistic regression model and ROC analyses

To assess the discriminating ability of the multiple logistic regression model, the AUC, along with the sensitivity and the specificity, was reported in a stepwise manner and presented in **Table 4**. The model with the covariates wrist circumference, gender, ALT, and TG can discriminate approximately 76.3% of the cases with high CAP scores. The AUC is presented in **Figure 1**. Furthermore, this model showed 69.4% sensitivity and 73.4% specificity, as detailed in **Table 4**. Finally, by applying the Youden index, the optimal cutoff points for wrist circumference were 18 and 16.3 cm for men and women, respectively.

**Table 4:** Stepwise analysis of the area under the ROC curve for predicting the odds of a high controlled attenuation parameter (CAP) score using the anthropometric and lipid covariates (*N* = 449).

Model/covariates included	Sensitivity^a^	Specificity^a^	AUC (95%CI)	Optimal cutoff point
Wrist circumference	0.778	0.459	0.654 (0.594–0.714)	Men = 18.0 Women = 16.3
Wrist circumference + gender	0.657	0.616	0.660 (0.599–0.722)	
Wrist circumference + gender + ALT	0.725	0.684	0.739 (0.682–0.797)	
Wrist circumference +gender+ ALT + TG	0.694	0.734	0.763 (0.709–0.818)	

*AUC*, area under the curve; *TG*, triglycerides; *ALT*, alanine transaminase.

a Both were calculated based on the optimal cutoff points for the classification obtained using the Youden index.

**Figure 3:**
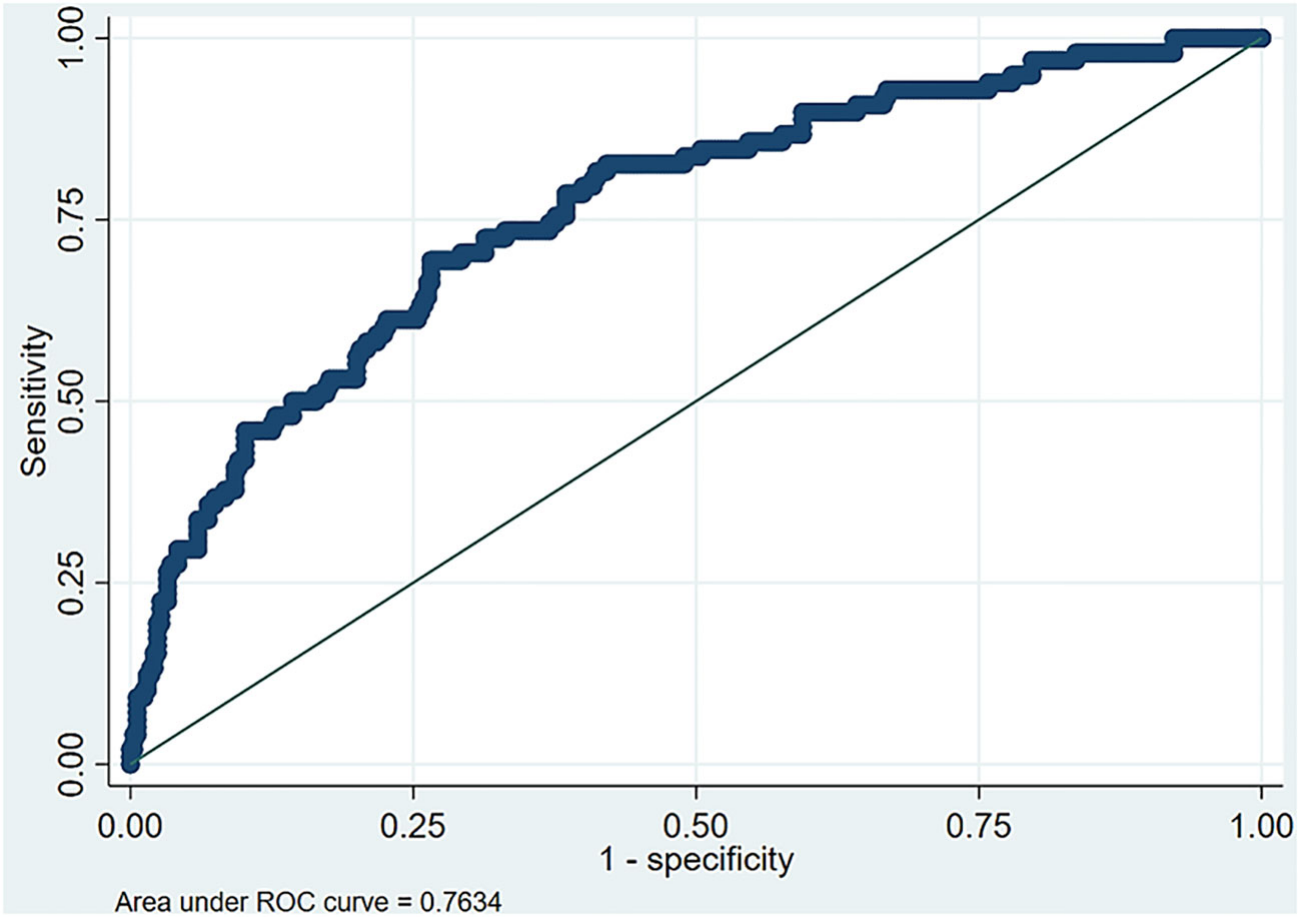
Area under the receiver operating characteristic (ROC) curve for the optimal multiple logistic regression model.

The ROC curve in **Figure 3** demonstrates a significant area between the curve of the predicted probability and the reference line, which shows the accuracy of increased wrist circumference between the participants with normal levels and the participants with MAFLD.

## 4 Discussion

Although several noninvasive diagnostic tools have been utilized to identify individuals at a higher risk of developing MAFLD, including blood-based biomarkers for steatosis and fibrosis, in addition to the bioindicators of MAFLD (14, 15), the correlation between wrist circumference and MAFLD has not been thoroughly explored. This study demonstrated that wrist circumference can be an effective noninvasive diagnostic tool for the identification of individuals at a higher risk of developing MAFLD by highlighting the significant variations in the wrist circumference values across different CAP scores. Our findings showed a progressive increase in the severity of hepatic steatosis, which is associated with other metabolic dysfunctions, including elevated TG, ALT, AST, and wrist circumference. In addition, wrist circumference was inversely correlated with HDL, supporting our initial hypothesis. Our results align with numerous published reports showing that the severity of hepatic steatosis and other metabolic dysfunctions negatively correlated with HDL (16–21). This underscores the critical role of anthropometric measurements in the clinical assessment of MAFLD. By integrating wrist circumference into the diagnostic process, healthcare professionals may enhance their ability to identify at-risk individuals, ultimately facilitating earlier interventions and the management of MAFLD and its associated health complications.

While BMI and waist circumference are well-established markers of adiposity and metabolic risk, they are also strongly correlated with wrist circumference. Increased adiposity and its metabolic consequences may contribute to the association between higher CAP scores and greater wrist circumference, which are predominant in the pathogenesis of MAFLD, as several published reports have presented a positive correlation between increased adiposity and the CAP score and wrist circumference (22–24). Our results indicated that, for every additional centimeter in wrist circumference, the likelihood of having a high CAP score rises by more than half compared with individuals with a smaller wrist circumference, indicating a substantially elevated risk of hepatic steatosis. Given the strong correlation between wrist circumference and MAFLD, we assessed its diagnostic potential using ROC analysis. The results demonstrated that wrist circumference has significant diagnostic value for the identification of individuals with MAFLD. The AUC was 0.763, indicating that this model has a discriminating predictive ability of 76.3% in differentiating between MAFLD patients and healthy individuals. These findings suggest that wrist circumference could be a promising novel biomarker for the assessment and prediction of the risk of developing MAFLD. Given that measuring the wrist circumference is a simple and cost-effective procedure, it has the potential to significantly enhance the early screening and diagnostic processes for MAFLD. This is particularly important in settings where access to more complex diagnostic tools, such as imaging techniques or advanced blood tests, may be limited or impractical.

While our study is among the first to demonstrate a link between wrist circumference and MAFLD, the dataset was limited to Kuwaiti participants. This may impact the generalizability of our findings to other populations with diverse ethnic and cultural backgrounds. Further studies are warranted to explore its potential to establish more definitive predictive models for clinical use. By expanding the background of the study population, researchers can assess the consistency and reliability of wrist circumference as a diagnostic tool for MAFLD. This will help determine whether the observed associations are accurate across diverse populations or if there is a need for population-specific thresholds or adjustments. In addition, data on the hepatitis status and medication use of the participants were not collected, which may have introduced residual confounding and should be addressed in future studies. Moreover, due to the cross-sectional study design, the current findings can be used to establish an association between wrist circumference and the CAP score, but not a causal effect. Furthermore, longitudinal studies are needed to evaluate the predictive value of wrist circumference in identifying individuals at risk of developing MAFLD and associated metabolic complications. This information is crucial for the development of evidence-based guidelines on the use of wrist circumference in clinical practice. One final note worth mentioning is that, in the multiple logistic regression, when the outcome is prevalent (23% in our case), the OR may overestimate the effect size. For this reason, the relative risk may be reported; thus, ORs should be interpreted with caution.

In conclusion, this study showed that wrist circumference may serve as a safe, noninvasive outpatient approach for the screening of individuals with an elevated risk of developing MAFLD that can be further confirmed with approved diagnostic procedures. This method enables early detection, is cost-effective, and reduces the need for unnecessary invasive testing. While wrist circumference alone may serve as a scalable and practical screening tool for MAFLD risk, its diagnostic utility could be substantially improved when combined with additional clinical or biochemical markers in future predictive models. Further research is necessary to validate its applicability across diverse populations and to establish its role in predictive models for clinical use.

## Data Availability

The raw data supporting the conclusions of this article will be made available by the authors, without undue reservation.
